# Assessment of Risk, Vulnerability and Adaptation to Climate Change by the Health Sector in Madagascar

**DOI:** 10.3390/ijerph15122643

**Published:** 2018-11-26

**Authors:** Norohasina Rakotoarison, Nirivololona Raholijao, Lalao Madeleine Razafindramavo, Zo Andrianina Patrick Herintiana Rakotomavo, Alain Rakotoarisoa, Joy Shumake Guillemot, Zazaravaka Jacques Randriamialisoa, Victor Mafilaza, Voahanginirina Anne Marie Pierrette Ramiandrisoa, Rhino Rajaonarivony, Solonomenjanahary Andrianjafinirina, Venance Tata, Manuela Christophère Vololoniaina, Fanjasoa Rakotomanana, Volahanta Malala Raminosoa

**Affiliations:** 1 Ministry of Public Health, Health and Environment Service, Antananarivo 101, Madagascar; norohasinarakotoarison@gmail.com (N.R.); razafindramavolalaomadeleine@gmail.com (L.M.R.); mafilaza57@gmail.com (V.M.); tata_ssenv@yahoo.fr (V.T.); 2 Madagascar National Meteorological and Hydrological Service, Antananarivo 101, Madagascar; yandrianina@yahoo.fr (Z.A.P.H.R.); voahangy_ramiandrisoa@yahoo.com (V.A.M.P.R.); rajaonarivony@gmail.com (R.R.); njafys@yahoo.fr (S.A.); 3 Ministry of Public Health, Direction of Health Surveillance and Epidemiological Surveillance, Antananarivo 101, Madagascar; arissoa@gmail.com; 4 Word Health Organization/World Meteorological Joint Office, Geneva 2300, Switzerland; jshumake-guillemot@wmo.int; 5 Independent Statistician, Antananarivo 101, Madagascar; zjrandriamialisoa@yahoo.fr; 6 Ministry of Public Health, Emergency, Epidemic and Disaster Response Service, Antananarivo 101, Madagascar; manuelachristophere@gmail.com; 7 Pasteur Institute in Madagascar, Antananarivo 101, Madagascar; fanja@pasteur.mg; 8 Word Health Organization, Madagascar Office, Antananarivo 101, Madagascar; raminosoav@who.int

**Keywords:** Madagascar, assessment, health, climate trends, climate projections, climate-sensitive diseases, vulnerability, adaptation

## Abstract

Madagascar is cited as one of the most vulnerable countries to the effects of climate change, with significant impacts to the health of its population. In this study, the vulnerability of Madagascar’s health sector to climate change was assessed and appropriate adaptation measures were identified. In order to assess climate risks, vulnerability and identify adaptation options, the Madagascar Ministry of Public Health as well as the National Meteorological and Hydrological Service worked in close collaboration with a team of local experts to conduct a literature review, field surveys, and analyses of current and future climate and health trends. Four climate-sensitive diseases of primary concern are described in the study: acute respiratory infections (ARI), diarrhea, malnutrition, and malaria. Baseline conditions of these four diseases from 2000 to 2014 show acute respiratory infections and diarrheal diseases are increasing in incidence; while incidence of malnutrition and malaria decreased over this period. To assess future impacts in Madagascar, this baseline information was used with climate projections for the two scenarios—RCP 4.5 and RCP 8.5—for the periods 2016–2035, 2036–2070 and 2071–2100. Future climate conditions are shown to exacerbate and increase the incidence of all four climate sensitive diseases. Further analysis of the exposure, sensitivity and adaptive capacity to the climate hazards suggests that the health sector in four regions of Madagascar is particularly vulnerable. The study recommends adaptation measures to improve the monitoring and early warning systems for climate sensitive diseases, as well as to reduce population vulnerability.

## 1. Introduction

Madagascar is among the most vulnerable countries to the adverse effects of climate change. The population of approximately 25 million inhabitants, is predominantly rural with high levels of poverty, and is exposed to many significant extreme weather and climate events each year. The country lacks readily available resources to adequately prepare for, respond to, and recover from the damages of such events on the health sector [1]. Between 1990 and 2013, the country recorded 63 major natural disasters, affecting at least 13 million people [2]. The drought triggered by the strong El Niño phenomenon in September 2015 created an unprecedented nutritional crisis and precarious situation in the greater south of Madagascar. Due to the prolonged and severe drought that lasted from 2015–2017, 1,144,000 people were food insure, of which 665,000 people (including 333,752 women and girls) experienced severe food insecurity, and 475,000 were moderately affected [3]. Damages to the health sector from natural disasters each year are significant and continue long after the event. For example, in 2008, 167 Basic Health Centers and 6 hospitals were damaged [4]. Infectious diseases, emerging and re-emerging diseases and non-communicable diseases remain the leading causes of morbidity and mortality in both adults and children in Madagascar. Many of these diseases are sensitive to climate conditions [5], and are expected to be exacerbated in the future as global temperatures rise, and extreme events such as droughts and floods become more severe and frequent. Exacerbating the effects of increased human exposure, the country’s infrastructure and urban areas, especially in coastal zones, have not been developed to cope with the effects of current and future climate events. This can result in significant impacts on livelihoods, food security, infrastructure, and other sectors related to economic development.

This study was conducted by the Government of Madagascar to assess the risks, vulnerability and identify adaptation options to improve the health sector’s preparedness for the impacts of climate change [6]. To identify appropriate adaptation measures, analysis of current and future trends in health and climate parameters was conducted, along with an assessment of the adaptive capacity and vulnerability of the health sector and of households in specific areas. Four climate-sensitive diseases were considered: acute respiratory infections (ARI), diarrheal diseases, malnutrition and malaria as they are among the top ten causes of morbidity seen in outpatient clinics in the Basic Health Centers (CSBs). Some of these diseases are also among the top ten causes of hospital mortality in Madagascar, including malnutrition, which remains among the leading causes of death in children.

Monthly health data from January 2000 to December 2014 was analyzed along with climate data from 1981 to 2014. Climate projections were derived from the Coupled Model Intercomparaison Project version 5 (CMIP5) products.

## 2. Materials and Methods

### 2.1. Study Design

The vulnerability and adaptation assessment followed the methodology proposed by World Health Organization [7]. The study was conducted over ten months in 2015. A multidisciplinary study team comprised of experts in key areas such as meteorology, public health, epidemiology, malaria, malnutrition supported the project. The assessment focused on regional scale vulnerability mapping, establishing a baseline of indicators for key climate sensitive health outcomes, and projecting possible future disease trends. As a government led study, participating experts included those that are highly involved in Madagascar’s national disease control and prevention activities. This ensured that the outcomes of the study would relate directly to the health sector’s programmatic opportunities. Four analyses were conducted for the study:

- Climate and health trends for 12 districts;
- Climate and health projections for six districts;
- Health and socio economic correlation study for six districts;
- Health sector vulnerability assessment for 22 regions.

### 2.2. Madagascar Climate

**Figure 1:**
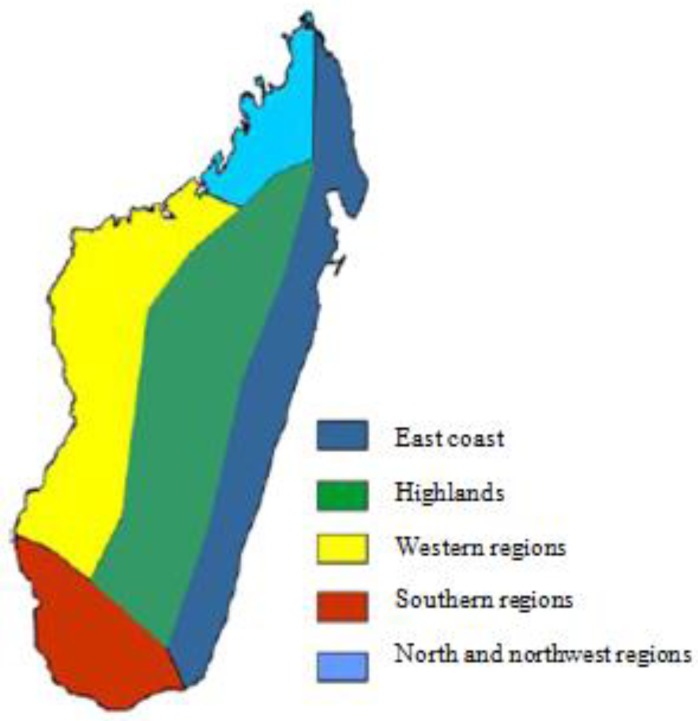
The five climate regions of Madagascar.

Madagascar is an island located 400 km from the southeast African coast. With an area of 587,041 km², Madagascar is the fourth largest island in the world. The geographical location, highly varied topography, maritime influence, and wind regimes explain the variety of climate and ecological regions on the island. Madagascar experiences two main seasons: a dry season from May to October, and a rainy season from November to April. Broadly, Madagascar is divided into five climate regions (Figure 1) [8,9,10]:

- The eastern coast has a warm and humid tropical climate;
- The central highland has a high altitude tropical climate;
- The western region has a warm climate and two distinct seasons, a dry winter and a hot and humid summer;
- The north and northwest region has a tropical climate, with the northwest monsoon conditions driving rainfall during the summer season;
- The southern region of the country has a semi-arid climate.

The main climate-related hazards affecting Madagascar are tropical cyclones, floods and droughts. On average, from November to April, three to four tropical cyclones hit Madagascar every year. Most cyclones form in the Indian Ocean, but some occasionally form in the Mozambique Channel and arrive from the west. All regions in Madagascar can be affected by tropical cyclones but the most affected are the eastern, northwestern, and western regions.

Tropical cyclones bring torrential flooding which often lead to dam breaks, as was the case in Fiherenena in 2013 after tropical cyclone Haruna struck the southwest region. Episodes of heavy summer rainfall can also cause flooding in cities, including the urban slums of Antananarivo, due to the lack of efficient rainwater drainage systems.

Droughts occur mostly in the southern regions of Madagascar which has a semi-arid climate, but it can also affect the central highlands and the eastern region. When the onset of the rainy season is delayed and the amount of precipitation is well below normal in the central highlands and eastern regions, rice production which is the staple of the local diet, is significantly affected.

### 2.3. Climate Hazards and Health Outcomes

The climate hazards used in the assessment were chosen according to three criteria:

- affecting Madagascar;
- generating significant negative socio-economic impacts, particularly on health;
- could be aggravated by climate change.

According to these criteria, three climate hazards—tropical cyclones, floods and drought—were selected for assessment within the exposure component of the vulnerability study. The health outcomes of interest are malnutrition, ARI, diarrhea, and malaria. They each: (i) have established associations with climate parameters, (ii) constitute a public health problem in Madagascar, (iii) exhibit morbidity rates that can be reduced by preventive interventions, and (iv) have data of sufficient quality to conduct longitudinal analysis.

### 2.4. Target Regions and Districts

**Figure 2:**
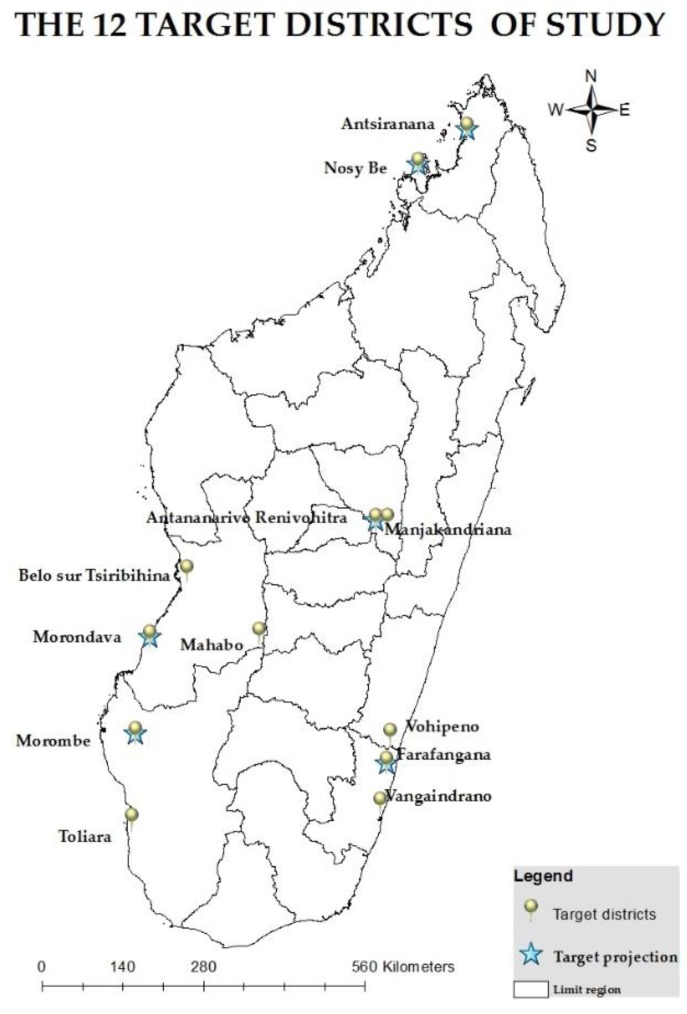
The target districts for the assessment of climate and health trends.

Based on the five climate regions of Madagascar (eastern coast, central highland, western region, north and northwest region and southern region), the availability of climate projections, meteorological, and epidemiological data and considering the most vulnerable regions to the climate hazards as defined in the National Contingency Planon Cyclone and Floods [11], the study areas selected for the assessment were:

- For the health sector vulnerability study: 22 regions;
- For the study of climate and health trends: 12 districts (Antananarivo, Manjakandriana, Antsiranana, Nosy Be, Morondava, Belo on Tsiribihina, Mahabo, Toliara I, Morombe, Farafangana, Vangaindrano, Vohipeno) (Figure 2);
- For the study of climate projections and the study of current and future climate change and health burdens: six localities (Antsiranana, Nosy Be, Antananarivo, Morondava, Farafangana, Morombe). These six localities were also used for the correlations study between climate-sensitive diseases and climate and socio-economic indicators.

The unit of analysis for the vulnerability mapping was the region since available databases capture data by region and allowed for an aggregate perspective of vulnerability conditions.

### 2.5. Data Sources

#### 2.5.1. Weather and Climate Data

Weather and climate data were obtained from the Madagascar National Meteorological and Hydrological Service (MNMHS). For study sites without observation stations, data from the MNMHS’ENACTS (Enhancing National Climate Services) database was used, which merges data from the national station with remote sensing products.

The data for the 12 districts included monthly series of precipitation, number of rainy days, and maximum and minimum temperatures from 1984 to 2014. Projected climate data were obtained from 11 global climate models in the fifth phase of CMIP5 corresponding to the Fifth Intergovernmental Panel on Climate Change (IPCC) Assessment Report (AR5) emission scenarios RCP 4.5 and RCP 8.5 (Available from the University of Cape Town Climate Information Portal, http://cip.csag.uct.ac.za). The climate parameters are monthly precipitation, minimum and maximum temperatures for the three time-slices of 2016–2035, 2036–2070 and 2071–2100.

#### 2.5.2. Epidemiological Data

The Ministry of Public Health provided epidemiologic data from 2000–2014 that were obtained from the National Statistical Branch, including the number of monthly cases and rate of primary health care visits for ARIs, malaria, malnutrition and diarrhea for each target district.

#### 2.5.3. Socio-Economic Data

Based upon the demographic and health survey of 2008–2009 [12], the National Statistical Institute of Madagascar provided information on household poverty rates, the percentage of active men working in agriculture, the rate of accessibility to water, sanitation and hygiene (WASH) infrastructure, accessibility to the nearest health facility, the percentage of housing with walls built of materials resistant to climate hazards, demographic data, health status and communication conditions, literacy rates, non-farm business ownership, and average household annual income.

### 2.6. Statistical Methods

Simple linear regression and trend analyses were conducted for the health data.

For the projection of health trends, SPSS software (version 20, SPSS Inc., Chicago, IL, USA) was used to build a multivariate statistical model using rainfall, minimum temperature and maximum temperature as independent variables:

Y = A_0_ + A_1_·X_Rain_ + A_2_·X_Tmax_ +A_3_·X_Tmin_ (1)

where Y = number of disease cases, A_0_ = constant, A_1_ = regression coefficient for rainfall, A_2_ = regression coefficient for maximum temperature, A_3_ = regression coefficient for minimum temperature, X_Rain_ = projected value of rainfall, X_Tmax_ = projected value for maximum temperature, and X_Tmin_ = projected value for minimum temperature.

The following formula was employed to assess vulnerability [7]:

(2) $$\mathrm{Vulnerability}\;=\;\frac{\mathrm{Exposure}\;\times \;\mathrm{Sensitivity}}{\mathrm{Adaptive}\;\mathrm{capacity}}\;$$

Exposure at the household-level was measured by estimating exposure to cyclones, floods, and droughts. Sensitivity was measured using household economic indicators, such as accessibility to health infrastructure, housing characteristics, demographic conditions, health status and access to mobile phone networks. The adaptive capacity of the health system, the communities and households was measured using the ratio of health workers to population in the study area, the ratio of population served by basic health facilities, the geographic accessibility rate, literacy rates, the non-farm business ownership rates and the average annual income. The identification of adaptation options to reduce health risks was based on expert judgment retrieved during the focus groups that were held in each target district. For the analysis of climate trends, the Mann Kendall statistical test and linear regression analysis were used to identify whether the climate trends are significant variables for the occurrence and distribution of the climate sensitive diseases of interest.

### 2.7. Limits of the Study

The assessment of regional vulnerability to the health risks posed by climate change was based on secondary data and the indicators selected depended on their availability. The regionally aggregated data are representative at a regional scale, however strong differences in data quality are observed when considered at district and commune level. In addition, the data were collected over different time periods. Epidemiological data from the Ministry of Public Health is collected through routine surveillance conducted by health facilities. However, national average attendance rates for basic health facilities is low at around 40%, and varies greatly among regions and districts, suggesting that official sources may underestimate the actual prevalence and conditions of many climate sensitive diseases.

## 3. Results

### 3.1. Climate Trends and Projections in the Target Districts

For the analysis of temperature trends, minimum temperatures in the winter season (June—July—August) and maximum temperatures in the summer season (December—January—February) were considered since these climate extremes influence the transmission mode of climate sensitive diseases. Early summer (October—November), core summer (December—January—February) and late summer (March—April) seasonal precipitation rates were analyzed as they determine the starting and ending dates of many disease epidemics such as plague and malaria.

#### 3.1.1. Climate Trends in the 12 Target Districts

The trends and their statistical significance at the 95% confidence level were completed for the climate data from 1984 to 2014. Warming trends in the maximum and minimum annual temperatures were observed across all 12 districts. The winter minimum temperatures (June—July—August) demonstrate warming trends for Farafangana and Vohipeno while the summer maximum temperatures (December—January—February) demonstrate warming trends for Antananarivo and Manjakandriana [13].

Trends in annual precipitation for Antsiranana, Farafangana, Vohipeno and Vangaindrano suggest a decrease in precipitation. No significant trends were observed elsewhere. Trends in rainy season precipitation (December—January—February) also suggest a decreasing trend for Antsiranana, Farafangana and Vohipeno. There were no significant trends observed for the onset (October—November) and the end of the rainy season (March—April) across the 12 districts [13].

#### 3.1.2. Climate Projections for the 6 Target Districts

Winter minimum temperatures (June—July—August) and the summer maximum temperatures (December—January—February) are projected to be warmer than the normal values (1971–2000) across all 6 districts under the two emission scenarios RCP 4.5 and RCP 8.5 and over the three time-slices 2016–2035, 2036–2070 and 2071–2100. Core summer season (December—January—February) precipitation is projected to slightly decrease compared to normal values under the two emission scenarios and over the three time-slices, except for Antsiranana where it is projected to slightly increase for 2016–2035 and 2036–2070. Early summer (October—November) precipitation is projected to slightly increase under the two emission scenarios, except for Farafangana where it is projected to slightly decrease. Late summer season (March—April) precipitation is projected to increase, except for Antsiranana and Farafangana where it is projected to decrease [13].

### 3.2. Trends and Projections of Health Parameters

#### 3.2.1. Observed Health Trends

**Figure 3:**
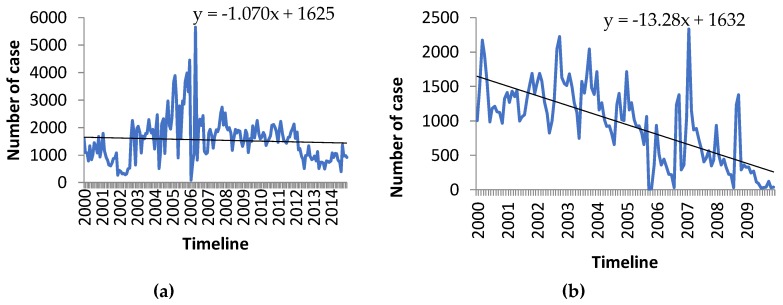
Observed trends of (**a**) malnutrition in the city of Antananarivo and (**b**) malaria in Morondava.

**Figure 4:**
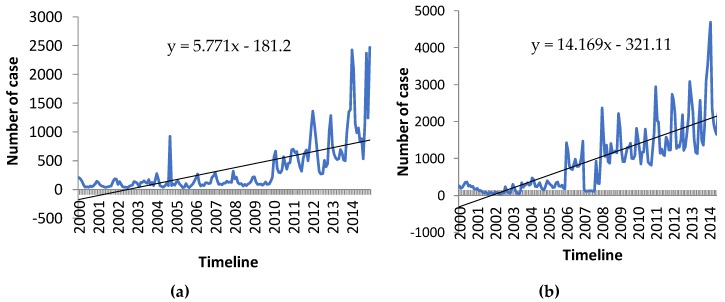
Observed trends of (**a**) diarrhea in Antsiranana and (**b**) ARI in Farafangana.

Figure 3 and Figure 4 display examples of observed trends in four selected localities for malnutrition, malaria, ARIs and diarrheal diseases, which are among the main reasons for use of health facilities by the population. In Figure 3, malnutrition in Antananarivo has a slightly decreasing trend from the year 2000 to 2014 and malaria in Morondava shows a decreasing trend from January 2000 to December 2009.

In Figure 4, ARI in Farafangana and diarrhea in Antsiranana both demonstrate an increase in observed trends.

#### 3.2.2. Health Projected Trends

**Figure 5:**
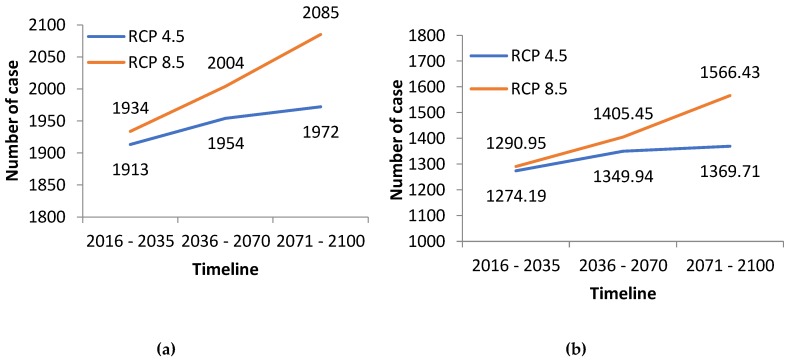
Projected trends of (**a**) Malnutrition in Antananarivo and (**b**) Malaria in Morondava.

**Figure 6:**
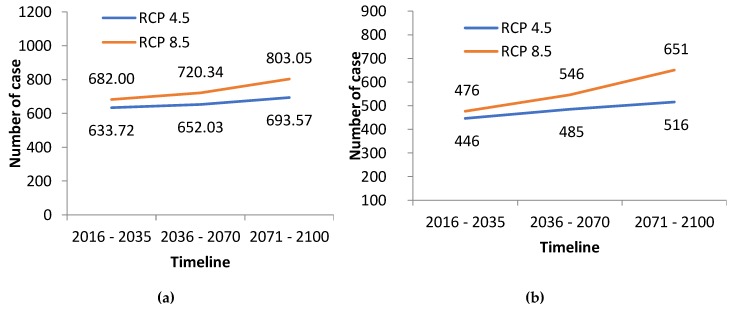
Projected trends of (**a**) ARI in Farafangana and (**b**) Diarrhea in Antsiranana.

The results from the multiple regression projection (Figure 5 and Figure 6) indicate an increase in projected trends for malnutrition in Antananarivo and for the three climate sensitive diseases in the other localities.

### 3.3. Correlations between Main Socioeconomic Factors and Climate Determinants for Cases of Climate-Sensitive Pathologies

Malagasy households exhibit many factors that increase their vulnerability to the health risks from climate variability and change (Table 1).

This study demonstrated statistically significant correlations (at different degrees) between the frequency of occurrence of climate-sensitive health outcomes, three key socio-economic determinants (literacy, access to drinking water, and poverty rates), and climate parameters (rainfall, minimum and maximum temperatures, and number of rainy days) (Table S1–S4).

Poverty and literacy rates are influential for all climate-sensitive health outcomes in almost all of the studied districts. Correlations between health outcomes and the five other variables were also observed, but the correlation significance varied by district.

### 3.4. Health Sector Vulnerability Mapping

**Figure 7:**
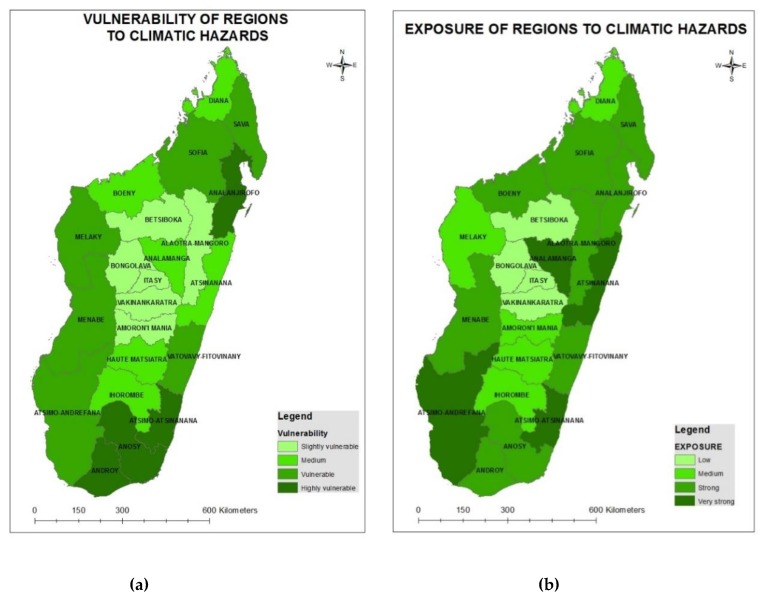
Vulnerability (**a**) and exposure (**b**) of regions in Madagascar to climate hazards.

**Figure 8:**
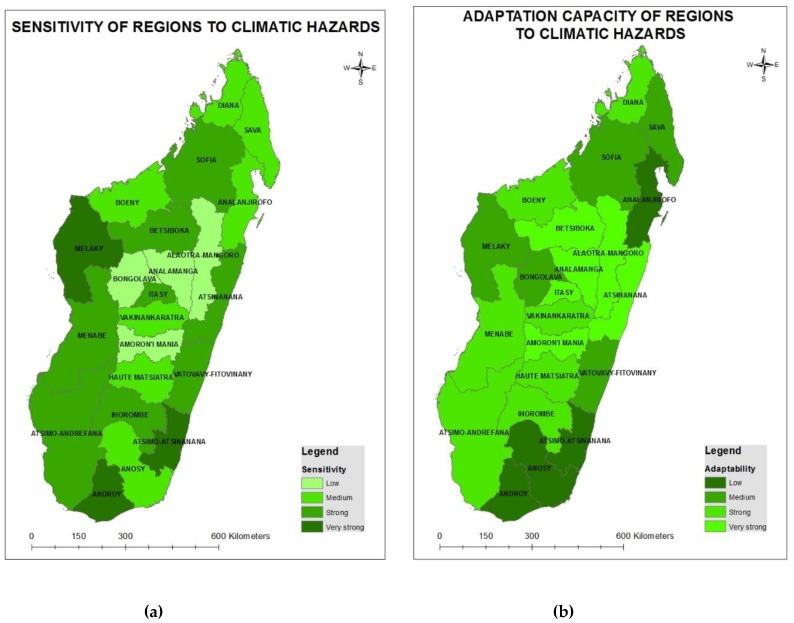
Sensitivity (**a**) and adaptive capacity (**b**) of regions in Madagascar to climate hazards.

The health status of the population and potential impacts from climate change are highly dependent on the aforementioned vulnerability factors. Regional levels of vulnerability (Figure 7a) to the health risks posed by climate change vary significantly across Madagascar, as observed by the differences in the level of exposure to climate hazards (cyclone, flood, drought) (Figure 7b), the level of sensitivity (baseline health status and socio-economic conditions) (Figure 8a), and the observed adaptive capacity (Figure 8b) of the health system, communities and households (see Table S5–S8).

The dark green color indicates regions that are most frequently affected by climate hazards and most sensitive to the health risks posed by the climate hazards. These regions also have a very low adaptive capacity and are the most vulnerable regions to climate change in Madagascar. Four out of 22 regions are highly vulnerable to the health impacts of climate change: Atsimo Atsinanana, Androy, Anosy and Analanjirofo.

### 3.5. Adaptation Options for the Health Sector

Madagascar has already taken important steps to address climate change through the development of a national health adaptation plan [14] and the publication of the Madagascar Climate and Health Profile [15]. However, due to evidence of the link between some diseases, climate and socioeconomic factors—and higher levels of vulnerability to the health risks of climate change in some regions, the following adaptation measures should be implemented:

(1) Integrate climate risk management into health system activities, particularly in program and strategic areas of public health:
  - Early warning systems for climate and health and;
  - Building health system adaptive capacity (including disaster response capacity)
(2) Strengthen community resilience:
  - Create a common vision on how to reduce the health risks from climate change;
  - Integrate adaptation measures into development plans at all levels: region, district, commune, fokontany;
(3) Mobilize local actors to:
  - Strengthen community solidarity;
  - Support local development;
  - Improve climate change risk management sensitization;
(4) Reduce the vulnerability of households by:
  - Improving the standard of living across the population;
  - Diversifying income sources;
  - Improving living conditions such as habitats, access to basic social services, food security, social protection and security.

## 4. Discussion

The decreasing trend in the prevalence of malaria in Morondava (Figure 3b) and of malnutrition in the city of Antananarivo (Figure 3a) may be explained by high levels of investment in malaria control and in food and nutritional security over the study period. Nevertheless, the assessment suggests projected increases in the prevalence of these two health outcomes in the future due to the impacts of climate change (Figure 5). For example, it is well known that decreases in rainfall reduces the availability of surface and ground water and can lead to reduced agricultural production, animal survival and nutrition among the population, ultimately leading to poorer health. In Madagascar, the negative impacts of drought on crop yields and subsequently on the nutritional status of the population have been observed frequently in the past. These events have resulted in the loss of life and livelihoods and have also undermined development investments in economic and nutritional security within the region.

For malaria, the projected increase in cases can be explained by the expected expansion of the mosquito vector’s habitat range to areas where it does not currently exist. This aligns with the IPCC Fifth Assessment Report which reported a change in the geographical distribution of some vector-borne diseases [16]. Multiple microclimates (tropical hot and humid, semi-humid, semi-arid), the expected increased variability of precipitation and the continuous increases in temperature can modify the number and the quality of mosquito vectors’ larval deposits, as well as vegetation density, and influence the resting spots for mosquitos. A high density of vectors, combined with decreasing rates of immunity due to previous control interventions, or a possible reduction in the effectiveness of future control measures, could trigger disease outbreaks and expand transmission. If the most significant effects of climate change on disease transmission are likely to occur at the extremes of the temperature range by favoring the transmission of many diseases, (generally 14–18 °C for the lowest temperatures and 35–40 °C for the highest temperatures [17]), the projected increase in the average annual temperature of Madagascar (currently between 14 °C and 27.5 °C depending on region) will result in many areas becoming more favorable to the transmission of climate-sensitive vector-borne diseases, including malaria, as well as dengue, chikungunya, and yellow fever. These findings are supported by a study commissioned by the WHO showing that the risk of vector-borne diseases, such as malaria and dengue, are expected to increase in Madagascar towards 2070 [15].

In addition, ARIs in Farafangana (Figure 4b) and diarrheal diseases in Antsiranana (Figure 4a) show an increasing observed trend from 2000–2014 and both are also expected to increase in the future as the climate continues to change. ARIs, with a morbidity rate of 30%, are by far the leading reason for visits to basic health facilities in Madagascar. In addition to climate factors, the poor quality of ambient air (due to industrial pollution, deforestation, burning of waste and indoor air pollution from biomass burning for cooking and heating), poor nutritional status are all risk factors for the occurrence of ARIs. Further studies are required to determine the reasons behind the continued increase in the prevalence of ARIs in Madagascar and to identify which risk factors are most significant.

Significant correlations of varying degrees were found between the frequency of climate-sensitive health outcomes, socio-economic determinants and climate parameters. This finding suggests important relationships exist between climate conditions and socio-economic factors of households that are important to the health status of Madagascar’s population in specific localities as well as a whole. Differences in exposure and vulnerability also result from non-climate stressors and multidimensional inequalities among the population that are often produced by unequal development processes. These differences help to determine differential health risks due to climate change. Ultimately, Madagascar’s 22 regions show different levels of vulnerability to the health risks posed by climate change. Areas with high levels of exposure and low adaptive capacity are among the most vulnerable, as is the case in Farafangana. Poverty and low literacy rates appear to be influencing factors for all health outcomes of interest studied within this assessment.

To better understand and guide decision-making processes that support planning, monitoring, preparedness, and implementation of control measures through adaptation, further analyses and modeling of these health risks with climate change scenarios is needed while also considering Madagascar’s the diverse geography, climate zones, and socio-economic conditions.

## 5. Conclusions

The findings of this assessment will serve to enhance knowledge on the link between climate change and diseases of concern in Madagascar and will inform actions taken by the health sector to protect the population from the health risks of climate change.

This study revealed two striking results:

- The population of Madagascar and the health sector are already very vulnerable to the risks of current climate variability as evidenced by observations of current health impacts;
- Climate change is expected to increase risks to health and well-being in the future.

Strengthening the adaptive capacity of the population and of the health sector is an urgent need and actions should be taken to empower the population, especially in more vulnerable areas, to adopt needed protective measures.

Continuous collaboration between the health and meteorological sectors is essential. The health sector should use current and projected climate data to inform the development of a climate and health early warning system.

The reduction of vulnerability at the household-level also represents an important adaptation measure to reduce negative health impacts from climate change. It is therefore essential to implement a rigorous system and a national action plan to guide appropriate and effective household adaptation actions and increase Madagascar’s resilience to climate change.

## Figures and Tables

**Table 1:** Household vulnerability factors in Madagascar.

Category of Vulnerability	Vulnerability Factor
Economic vulnerability	Low income Livelihood dependent on natural resources
Social vulnerability	High child malnutrition Area prone to communicable diseases Low accessibility to health infrastructure Limited access to water and sanitation Low literacy
Environmental vulnerability	Precarious housing Hazardous area Remoteness

## Supplementary Materials

The following are available online at http://www.mdpi.com/1660-4601/15/12/2643/s1, Table S1: Correlation between diarrhea and climate and socioeconomical parameters, Table S2: Correlation between malnutrition and climate and socioeconomical parameters, Table S3: Correlation between malaria and climate and socioeconomical parameters, Table S4: Correlation between ARI and climate and socioeconomical parameters, Table S5: Matrix of the exposure assessment, Table S6: Matrix of sensitivity assessment, Table S7: Matrix of adaptive capacity assessment, Table S8: Matrix of the regions’ vulnerability assessment.
