# Supplementary material for: Assessment of Risk, Vulnerability and Adaptation to Climate Change by the Health Sector in Madagascar
Source: Int J Environ Res Public Health. 2018 Nov 26;15(12):2643. doi: 10.3390/ijerph15122643 (PMC6313613; doi:10.3390/ijerph15122643)
Supplement: Supplementary file 1 [file ijerph-15-02643-s001.pdf]

International Journal of
Environmental Research and Public Health

MDPI

Supplementary Material

Assessment of Risk, Vulnerability and Adaptation to Climate Change by the Health Sector in Madagascar

Norohasina Rakotoarison [1], Nirivololona Raholijao [2,*], Lalao Madeleine Razafindramavo [1], Zo Andrianina Patrick Herintiana Rakotomavo [2], Alain Rakotoarisoa [3], Joy Shumake Guillemot [4], Zazaravaka Jacques Randriamialisoa [5], Victor Mafilaza [1], Voahanginirina Anne Marie Pierrette Ramiandrisoa [2], Rhino Rajaonarivony [2], Solonomenjanahary Andrianjafinirina [2], Venance Tata[1], Manuela Christophère Vololoniaina [6], Fanjasoa Rakotomanana [7] and Volahanta Malala Raminosoa [8]

Table S1. Correlation between diarrhea and climate and socioeconomical parameters.

DISTRICT	DIARRHEA	TMIN	TMAX	Rainfall	Rainyday	Water	ALPHABETIZATION	O Poverty
ANTANANARIVO	Pearson Corr	0.339**	0.423**	0.184*	-0.090	0.063	-0.455**	-0.318**
	Sig. (bilatérale)	0.000	0.000	0.014	0.243	0.403	0.000	0.000
DIEGO SUAREZ I	Pearson Corr	0.146	0.195**	-0.009	0.074	0.367**	-0.539**	-0.560**
	Sig. (bilatérale)	0.051	0.009	0.905	0.321	0.000	0.000	0.000
FARAFANGANA	Pearson Corr	0.134	-0.033	-0.002	0.051	0.368**	0.643**	0.600**
	Sig. (bilatérale)	0.073	0.657	0.981	0.515	0.000	0.000	0.000
MOROMBE	Pearson Corr	0.151*	0.122	0.187*	0.217**	0.049	0.374**	-0.516**
	Sig. (bilatérale)	0.043	0.101	0.012	0.007	0.515	0.000	0.000
MORONDAVA	Pearson Corr	0.271**	0.088	0.324**	0.418**	0.161*	0.347**	-0.513**
	Sig. (bilatérale)	0.000	0.238	0.000	0.000	0.031	0.000	0.000
NOSY BE	C Pearson Corr	0.126	-0.092	-0.051	-0.098	0.091	-0.703**	-0.741**
	Sig. (bilatérale)	0.093	0.221	0.494	0.189	0.223	0.000	0.000

Table S2. Correlation between malnutrition and climate and socioeconomical parameters.

DISTRICT	MALNUTRITION	TMIN	TMAX	Rainfall	Rainy day	Water	ALPHABETIZATION	Poverty
ANTANANARIVO	Pearson Corr	0.122	0.183*	0.067	0.166*	-0.354**	0.015	0.166*
	Sig. (bilatérale)	0.104	0.014	0.375	0.030	0.000	0.837	0.026
ANTSIRANANA I	Pearson Corr	0.183*	0.117	0.187*	0.198**	-0.296**	0.112	0.063
	Sig. (bilatérale)	0.014	0.116	0.012	0.008	0.000	0.136	0.398
FARAFANGANA	Pearson Corr	0.011	0.126	0.223**	0.134	0.218**	-0.268**	0.068
	Sig. (bilatérale)	0.884	0.093	0.003	0.084	0.003	0.000	0.364

MOROMBE	Pearson Corr	0.158*	0.168*	0.021	0.139	0.091	0.133	0.364**
	Sig. (bilatérale)	0.034	0.024	0.782	0.085	0.226	0.075	0.000
MORONDAVA	Pearson Corr	0.236**	0.183*	0.223**	0.215**	-0.042	0.083	-0.028
	Sig. (bilatérale)	0.001	0.014	0.003	0.004	0.579	0.267	0.711
NOSY BE	Pearson Corr	0.028	-0.033	0.110	0.077	0.005	0.242**	0.235**
	Sig. (bilatérale)	0.707	0.663	0.141	0.305	0.948	0.001	0.002

Table S3. Correlation between malaria and climate and socioeconomical parameters.

DISTRICT	MALARIA (1 month)	TMIN	TMAX	Rainfall	Rainy day	Water	ALPHABETIZATION	Poverty
ANTANANARIVO	Pearson Corr	0.137	-0.006	0.160*	0.145	0.082	0.383**	0.116
	Sig. (bilatérale)	0.067	0.939	0.033	0.059	0.273	0.000	0.123
DIEGO SUAREZ I	Pearson Corr	0.100	0.008	0.227**	0.206**	-0.019	0.538**	0.680**
	Sig. (bilatérale)	0.184	0.918	0.002	0.006	0.804	0.000	0.000
FARAFANGANA	Pearson Corr	0.176*	-0.033	0.063	0.151	0.288**	0.046	0.310**
	Sig. (bilatérale)	0.018	0.657	0.400	0.053	0.000	0.542	0.000
MOROMBE	Pearson Corr	0.070	0.082	0.109	0.104	0.131	0.356**	0.488**
	Sig. (bilatérale)	0.354	0.277	0.148	0.200	0.080	0.000	0.000
MORONDAVA	Pearson Corr	0.115	0.153*	0.193**	0.007	0.159*	0.252**	0.586**
	Sig. (bilatérale)	0.127	0.041	0.010	0.931	0.033	0.001	0.000
NOSY BE	Pearson Corr	0.054	0.088	0.207**	0.211**	-0.021	0.425**	0.490**
	Sig. (bilatérale)	0.475	0.241	0.005	0.005	0.778	0.000	0.000

Table S4. Correlation between ARI and climate and socioeconomical parameters.

DISTRICT	ARI	TMIN	TMAX	Rainfall	Rainy day	Water	ALPHABETIZATION	Poverty
ANTANANARIVO	Pearson Corr	-0.028	0.096	-0.033	-0.215**	0.055	-0.652**	-0.161*
	Sig. (bilatérale)	0.708	0.201	0.658	0.005	0.464	0.000	0.031
DIEGO SUAREZ I	Pearson Corr	0.183*	0.218**	-0.036	-0.025	-0.077	-0.673**	-0.627**
	Sig. (bilatérale)	0.014	0.003	0.628	0.743	0.306	0.000	0.000
FARAFANGANA	Pearson Corr	0.119	0.040	-0.212**	-0.130	0.422**	0.741**	0.492**
	Sig. (bilatérale)	0.112	0.593	0.004	0.094	0.000	0.000	0.000
MOROMBE	Pearson Corr	0.022	-0.006	0.060	0.089	0.154*	0.288**	-0.606**
	Sig. (bilatérale)	0.765	0.938	0.426	0.269	0.040	0.000	0.000
MORONDAVA	Pearson Corr	0.174*	0.034	0.150*	0.373**	-0.035	0.127	-0.674**

	Sig. (bilatérale)	0.020	0.650	0.046	0.000	0.641	0.089	0.000
NOSY BE	Pearson Corr	0.118	0.054	-0.008	-0.086	0.014	-0.697**	-0.687**
	Sig. (bilatérale)	0.036	0.711	0.047	0.081	0.012	0.000	0.000

Table S5. Matrix of the exposure assessment.

**Exposure**	**Cyclone**<br>**Frequency from 2006 to 2014 (9 years, 24 cyclones)**	**Flood**<br>**Frequency from 1975 to 2014 (26 episodes)**	**Drought**<br>**Frequency of 1981 - 2014**<br>**(9 episodes)**
Very strong	Frequency> 8	Frequency>13	Frequency> 8
**Strong**	Frequencyfrom 5 to 8	Frequency from 5 to 13	Frequency from 5 to 8
**Average**	Frequencyfrom 3 to 5	Frequency from 1 to 5	Frequency from 1 to 5
**Low**	Frequencyof cyclone < 3	Frequency =0	Fréquency =0

Table S6. Matrix of sensitivity assessment.

**Sensitivity**	**Economic characteristics**	**Accessibility to health center(HC)**	**Characteristics of the habitat**	**Demographic, health and communication conditions**
**Very strong**	Poverty rate> 80% or Percentage of active men working in agriculture> 80%	Accessibility rate in WASH infrastructure <20% Or percentage of households making a journey of more than one hour to reach, from the town center, the nearest HC> 80%	Percentage of habitats with non-solid wall> 80%	Density of the population> 100 inhabitants / km2<br>Prevalence ARI in children under 5> 20%<br>Immunization rate of children aged 12 - 23 months <40%<br>Percentage of children aged 12 to 23 months with acute malnutrition> 10%
**Strong**	Poverty rate of 75 to 80% or Living percentage of natural resources 75% to 80%	Accessibility rate in WASH infrastructure from 20 to 40% Or households making a journey more than one hour to reach, from the town center, the	Percentage of dwellings in non-solid materials 60 to 80% Hazardous area (coastal, flood, arid, altitude> 800m - plague)	Density of the population from 50 to 100 inhabitants / km2<br>Prevalence ARI in children under 5 years of age from 15 to 20%<br>Immunization rate of children from 12 to 23 months from 40 to 60%<br>Percentage of children aged 12 to 23 months with acute malnutrition from 8.6% to 7%

Sensitivity	Economic characteristics	Accessibility to health center(HC)	Characteristics of the habitat	Demographic, health and communication conditions
		nearest HC 60 to 80%		
**Average**	Poverty rate of 65 to 75% or Percentage living of natural resources from 65% to 75%	Accessibility rate in WASH infrastructure from 40 to 60% Or percentage of population living more than 10km from 40 to 60% HC	Percentage of dwellings in non-solid materials from 40 to 60% Hazardous area (coastal, flood, arid, altitude> 800m - plague)	Density of the population from 25 to 50hab / km2 Prevalence IRA in children under 5 years of age from 10 to 15% Immunization rate of children from 12 to 23 months from 60 to 80% Percentage of children aged 12 to 23 months with acute malnutrition 5% to 7%
**Low**	Poverty rate of <65% or Living percentage of natural resources <65%	Percentage of households who hygienically remove stool from their children> 60% Or households making a journey more than one hour to reach, from the town center, the nearest HC<40%	Percentage of dwellings in non-solid materials <40% Hazardous area (coastal, flood, arid, altitude <800m - plague)	Density of the population of <25 inhabitants / km2 Prevalence IRA in children <5 years <10% Immunization rate of children from 12 - 23 months> 80% Percentage of children aged 12 to 23 months with acute malnutrition <5%

Table S7. Matrix of adaptive capacity assessment.

Adaptability	Health system	Community	Household
**Verystrong**	Physician ratio per 10,000 population> 1 Ratio of inhabitants per health facility <10,000	Lightweight car accessibility coverage rate permanently 100%	Literacy rate of individuals over 15 years> 80% Non-farm business ownership rate> 50% Average annual salary income> 2,000,000 Ar
**Strong**	Physician ratio per 10,000 inhabitants: 0.5 to 1 Ratio of inhabitants per health facility from 10,000 to 15,000	Coverage rate of communes in communication (mobile phone, Madagascar National Radio Channel(MNR,) light car	Literacy rate of individuals over 15 years of age from 70% to 80% Non-farm business ownership rate from 30% to 50% Average annual salary income from Ar 1,500,000 to Ar 2,000,000

Adaptability	Health system	Community	Household
		accessibility permanently) from 75 to 100%	
**Average**	Physician ratio per 10,000 inhabitants: 0.3 to 0.5<br>Ratio of inhabitants per health facility from 15,000 to 20,000	Municipal coverage rate in communication (mobile phone, MNR, light car accessibility permanently) from 50 to 75%	Literacy rate of individuals over 15 years of age from 50% to 70%<br>Non-farm business ownership rate from 25% to 30%<br>Average annual salary income from 1,200,000 Ar to 1,500,000 Ar
**Low**	Physician ratio per 10,000 population <0.3<br>Population ratio per health facility> 20,000	Communal coverage rate in communication (mobile phone, MNR, light car accessibility permanently) <50%	Literacy rate of individuals over 15 <50%<br>Non-farm business ownership rate <25%<br>Average annual salary income <1,200,000 Ar (SMIG)

Table S8. Matrix of the regions' vulnerability assessment.

REGION	EXPOSURE To hazards	SENSITIVITY to the health risks of hazards	ADAPTIVE CAPACITY*	VULNERABILITY
**East Coast**				
**Sava**				
**Analanjirofo**				
**Atsinanana**				
**VatovavyFitovinany**				
**AtsimoAtsinanana**				
**North West**				
**Diana**				
**Sofia**				
**Betsiboka**				
**Boeny**				
**Côte Ouest**				
**Melaky**				
**Menabe**				
**Central highlands**				
**Analamanga**				
**AlaotraMangoro**				
**Itasy**				

**Bongolava**				
**Vakinankaratra**				
**Ihorombe**				
**Amoron'i Mania**				
**Haute Matsiatra**				
**South**				
**Atsimoandrefana**				
**Androy**				
**Anosy**				

© 2018 by the authors. Submitted for possible open access publication under the terms and conditions of the Creative Commons Attribution (CC BY) license (http://creativecommons.org/licenses/by/4.0/).
